# Conductive Graphitic Carbon Nitride as an Ideal Material for Electrocatalytically Switchable CO_2_ Capture

**DOI:** 10.1038/srep17636

**Published:** 2015-12-01

**Authors:** Xin Tan, Liangzhi Kou, Hassan A. Tahini, Sean C. Smith

**Affiliations:** 1 Integrated Materials Design Centre (IMDC), School of Chemical Engineering, UNSW Australia, Sydney, NSW 2052, Australia

## Abstract

Good electrical conductivity and high electron mobility of the sorbent materials are prerequisite for electrocatalytically switchable CO_2_ capture. However, no conductive and easily synthetic sorbent materials are available until now. Here, we examined the possibility of conductive graphitic carbon nitride (g-C_4_N_3_) nanosheets as sorbent materials for electrocatalytically switchable CO_2_ capture. Using first-principle calculations, we found that the adsorption energy of CO_2_ molecules on g-C_4_N_3_ nanosheets can be dramatically enhanced by injecting extra electrons into the adsorbent. At saturation CO_2_ capture coverage, the negatively charged g-C_4_N_3_ nanosheets achieve CO_2_ capture capacities up to 73.9 × 10^13^ cm^−2^ or 42.3 wt%. In contrast to other CO_2_ capture approaches, the process of CO_2_ capture/release occurs spontaneously without any energy barriers once extra electrons are introduced or removed, and these processes can be simply controlled and reversed by switching on/off the charging voltage. In addition, these negatively charged g-C_4_N_3_ nanosheets are highly selective for separating CO_2_ from mixtures with CH_4_, H_2_ and/or N_2_. These predictions may prove to be instrumental in searching for a new class of experimentally feasible high-capacity CO_2_ capture materials with ideal thermodynamics and reversibility.

At the current rate of emissions of greenhouse gases, for which carbon dioxide (CO_2_) is the main component, global warming and climate change will continue to rise^1^,^2^,^3^. One crucial issue facing efficiently separating, capturing, storing and/or converting CO_2_ is the development of a practical sorbent material^4^,^5^,^6^. Liquid-amine, which is the most common adsorbent for current industrial process for CO_2_ capture, suffers from relatively low efficiency, equipment corrosion, solvent loss, and toxicity^7^,^8^,^9^,^10^. Alternatively, various solid materials have been proposed as attractive adsorbents for CO_2_ capture, including metal-organic frameworks (MOFs)^11^,^12^,^13^,^14^,^15^, aluminum nitride (AlN)^16^, carbon^17^,^18^,^19^, hexagonal boron nitride (*h*-BN)^20^, and silicon carbide (SiC)^21^,^22^ nanostructures. However, the difficult regeneration processes due to the large adsorption energy, which generally demands high temperatures to release captured CO_2_, significantly hinders their practical applications.

Recently, electrocatalytically switchable CO_2_ capture scheme has been proposed as a controllable, high selective, and reversible CO_2_ capture strategy for bare *h*-BN nanomaterials^23^. Specifically, CO_2_ molecules are weakly adsorbed (i.e. physisorbed) on neutral *h*-BN. By injecting extra electrons into *h*-BN adsorbent, density functional theory (DFT) calculations reveal that CO_2_ adsorption can be dramatically enhanced via a charge-induced chemisorption interaction. The chemically adsorbed CO_2_ can in principle be released when the extra electrons are removed. In contrast to previous methods, the CO_2_ capture/release occurs spontaneously once extra electrons are introduced or removed, and the process of CO_2_ capture/release can be simply controlled and reversed by switching on/off the charges carried by *h*-BN nanomaterials. However, *h*-BN is wide-gap semiconductor with band gap around 5.8 eV^24^,^25^ and it is not clear how to charge up bare *h*-BN due to its insulating character.

To overcome the above disadvantage, Jiao *et al*.^26^ have investigated carbon nanotubes with pyridinic-nitrogen as an alternative absorbent to electrocatalytically switchable CO_2_ capture because of their good electron conductivity. On the other hand, we have proposed layered *h*-BN and graphene (hybrid BN/G) nanosheets, consisting of a single or double-layer *h*-BN and a substrate graphene layer, as an experimentally feasible approach to induce the requisite charge on *h*-BN for electrocatalytically switchable CO_2_ capture^27^. However, the synthesis of carbon nanomaterials with pyridinic nitrogen doping and hybrid BN/G are difficult to control in experiment. One natural question arise: can we find a conductive sorbent material for electrocatalytically switchable CO_2_ capture, which avoids complicated synthesis route?

Very recently, intense attention has been attracted by a new class of two-dimensional conjugated polymer, graphitic carbon nitride, due to the anisotropic two-dimensional geometric morphology and the aromatic π-conjugated framework. This endows carbon nitride nanosheets with attractive bandgap- and surface-engineered applications in both energy- and environment-related topics, such as photocatalysis for water splitting^28^,^29^, hydrogen evolution^30^, CO2 reduction^31^, organosynthesis^32^, amongst others^33^.g-C_3_N_4_ and g-C_4_N_3_ are two kinds of two-dimensional conjugated nanosheets, which have been recently synthesized by using cross-linking nitride-containing anions in ionic liquid^34^,^35^. Different from each other, g-C_3_N_4_ is semiconductor^34^, while g-C_4_N_3_ shows half-metallic property^36^.

Here we show that electrocatalytically switchable CO_2_ capture is indeed possible by considering conductive g-C_4_N_3_ nanosheets, of which the charge states can be easily modified experimentally because of the good electrical conductivity and high electron mobility. Using first-principle calculations, we found that the adsorption energy of CO_2_ molecules on g-C_4_N_3_ nanosheets can be dramatically enhanced from 0.24 to 2.52 eV by injecting extra electrons into the adsorbent. At saturation CO_2_ capture coverage, the negatively charged g-C_4_N_3_ nanosheets achieve CO_2_ capture capacities up to 73.9 × 10^13^ cm^−2^ or 42.3 wt%. Once the extra electrons are removed, the captured CO_2_ molecules can easily desorb from the adsorbent. In contrast to other CO_2_ capture approaches, the process of CO_2_ capture/release occurs spontaneously without any energy barriers once extra electrons are introduced or removed, and these processes can be simply controlled and reversed by switching on/off the charging voltage. In addition, these negatively charged g-C_4_N_3_ nanosheets are highly selective for separating CO_2_ from mixtures with CH_4_, H_2_ and/or N_2_. These predictions might pave the way in searching for a new class of experimentally feasible high-capacity CO_2_ capture materials with ideal thermodynamics and reversibility.

## Results

**Figure 1:**
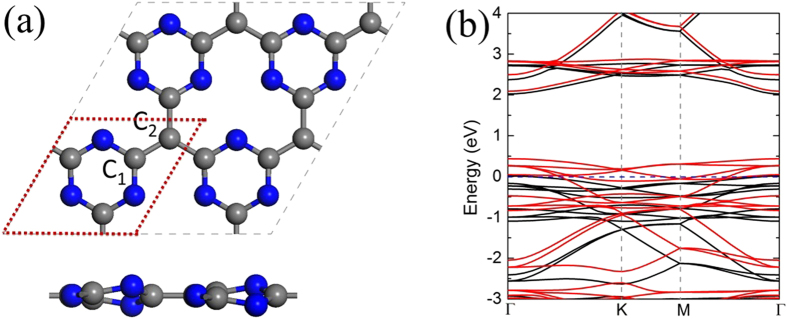
Top (upper) and side (lower) views of (**a**) a (2 × 2) reconstructed g-C4N3 supercell. The blue and grey balls represent N and C atoms, respectively, and the unit cell of g-C_4_N_3_ is indicated by red dot lines. C_1_ and C_2_ denote different C atoms in g-C_4_N_3_ unit cell. The calculated band structures of (**b**) a (2 × 2) reconstructed g-C_4_N_3_. The blue dashed line denotes the Fermi level. The red and black lines in (**b**) denote the spin-up and spin-down states, respectively.

Since good electrical conductivity and high electron mobility are prerequisite for injecting extra electrons into electrocatalytically switchable CO_2_ capture materials, we first studied the electronic structures of isolated g-C_4_N_3_. The lowest-energy configurations and the calculated band structures of g-C_4_N_3_ are shown in Fig. 1. Consistent with previous studies^36^, g-C_4_N_3_ is a (2 × 2) reconstructed structure with half-metallic state. This indicates that g-C_4_N_3_ has good electrical conductivity and high electron mobility, which should readily facilitate electron injection/release for electrocatalytically switchable CO_2_ capture.

### Single CO_2_ Adsorption on Neutral and 2 e^−^ Negatively Charged g-C_4_N_3_ Nanosheets

**Figure 2:**
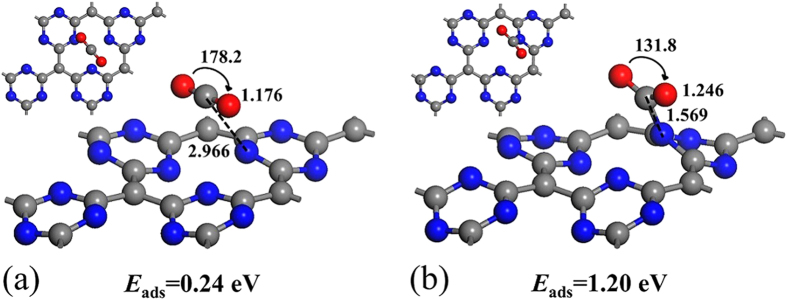
Top and side views of the lowest-energy configurations of a single CO_2_ molecule absorbed on the (**a**) neutral and (**b**) 2 e^−^ negatively charged g-C_4_N_3_. The blue, grey and red balls represent N, C and O atoms, respectively, and the adsorption energies of the CO_2_ molecule on neutral and 2 e^−^ negatively charged g-C_4_N_3_ are listed.

We next shift our attention to a single CO_2_ adsorption on neutral and negatively charged g-C_4_N_3_. Since g-C_4_N_3_ is a (2 × 2) reconstructed structure, there are many different adsorption sites for a CO_2_ molecule. Here, we considered all the adsorption sites: directly on top of a C or N atom, above the midpoint of a bond linking the C and N atoms, and above the center of a honeycomb-like hexagon. Figure 2 shows the lowest-energy configurations of a CO_2_ absorbed on neutral and 2 e^−^ negatively charged g-C_4_N_3_. On neutral g-C_4_N_3_ (Fig. 2(a)), the linear CO_2_ molecule is parallel to g-C_4_N_3_ and locates on top of three nitrogen atoms. The distance between the C atom of CO_2_ and closest N atom is 2.966 Å, and the linear CO_2_ molecule shows little structural change compared to a free CO_2_ molecule with the O-C-O angle and two double C=O bonds being 178.2° and 1.176 Å, respectively. Mulliken population analysis suggests that the amount of transferred electron from the absorbed CO_2_ molecule to g-C_4_N_3_ is negligible (about 0.004 e^−^). For the neutral case, the CO_2_ molecule is weakly adsorbed (i.e. physisorbed) onto neutral g-C_4_N_3_ with small adsorption energy of 0.24 eV.

After injecting two extra electrons into the g-C_4_N_3_ supercell (Fig. 2(b)), the CO_2_ is strongly adsorbed at surface N atom, and changes from physisorption into chemisorption on 2 e^−^ negatively charged g-C_4_N_3_. The distance between the C atom of CO_2_ and surface N atom of g-C_4_N_3_ is shortened to 1.569 Å, the O-C-O angle is bent from 178.2° to 131.8°, the two double C=O bonds are elongated from 1.176 to 1.246 Å, and the charge transfer from g-C_4_N_3_ to CO_2_ increase to 0.56e^−^. In this case, the adsorption energy of a CO_2_ remarkably increases to 1.20 eV, which is much larger than the adsorption energies of CO_2_ on other high-performance adsorbents (0.4–0.8 eV)^6^, indicating that the negatively charged g-C_4_N_3_ is an excellent adsorbent for CO_2_ capture.

**Figure 3:**
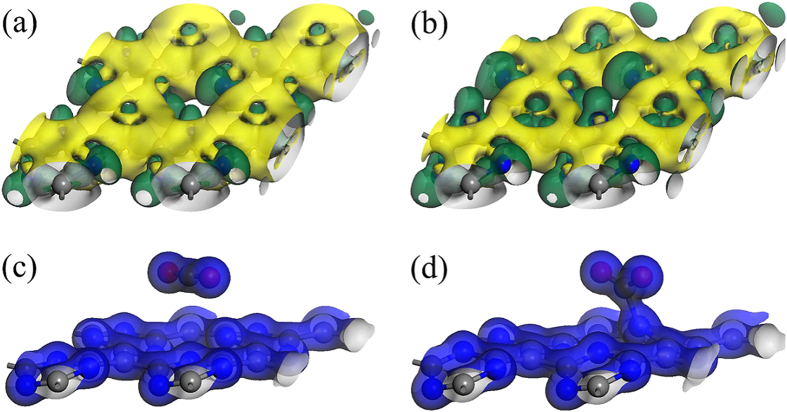
The deformation electronic density of (**a**) neutral and (**b**) 2 e^−^ negatively charged g-C_4_N_3_. Green and yellow refer to electron-rich and -deficient area, respectively. The isosurface value is 0.02 e/au. (**c**) The total charge density distribution of a single CO_2_ molecule on (**c**) neutral and (**d**) 2 e^−^ negatively charged g-C_4_N_3_. The isosurface value is 0.8 e/au. The overlap of the electron densities of the C atom of CO_2_ and surface N atom of g-C_4_N_3_ in (**d**) indicates the formation of a new bond.

To understand the underlying mechanism of CO_2_ capture on negatively charged g-C_4_N_3_, we plotted the deformation electronic density of neutral and 2 e^−^ negatively charged g-C_4_N_3_ by subtracting the electronic density of isolated N and C atoms from the sheet in Fig. 3. Obviously, for the neutral case (Fig. 3(a)), some electrons are extracted from the C atoms and delocalized over the N atoms, as implied by the green regions. Mulliken population analysis indicated that the electrons distribute at N, C_1_ and C_2_ are −0.302, 0.294 and −0.036 |e|, respectively. When two extra electrons are introduced (Fig. 3(b)), the extra electrons are almost evenly distributed on N and C atoms. Mulliken population analysis suggest that each atom gains −0.07 ~ −0.09 |e|, and the electrons distribute at N, C_1_ and C_2_ are −0.383, 0.222 and −0.122 |e|, respectively. Compared with the neutral case, more electrons are distributed and delocalized at N atoms, as implied by the green regions in Fig. 3(b). As CO_2_ is a Lewis acid and it prefers to accept, rather than donate, electrons during reaction, the N atom of negatively charged g-C_4_N_3_ can donate electrons to CO_2_, and form a new bond between the C atom of CO_2_ and surface N atom of g-C_4_N_3_ (Fig. 3(d)), which is significantly different from the case that CO_2_ on neutral g-C_4_N_3_ (Fig. 3(c). This is the reason why the CO_2_ molecule has a strong interaction with negatively charged g-C_4_N_3._

**Figure 4:**
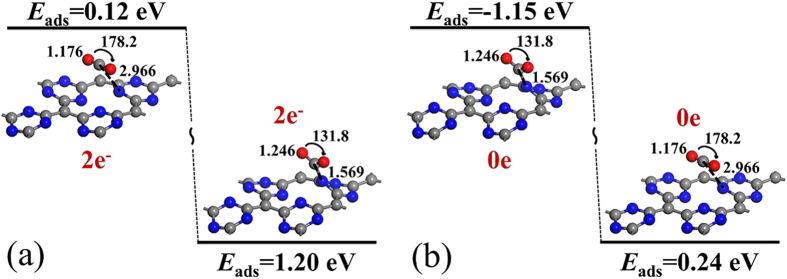
The energy change of (**a**) the relaxation (capture) of a CO_2_ molecule on g-C_4_N_3_ after two extra electrons are introduced, and (**b**) the reverse relaxation (release) process of a captured CO_2_ molecule from g-C_4_N_3_ after two extra electrons are removed from the adsorbent.

In order to investigate the kinetic process of CO_2_ capture/release on 2 e^−^ negatively charged g-C_4_N_3_, we next studied the energy change of a CO_2_ molecule adsorbed on g-C_4_N_3_ after the introduction or removal of the two extra electrons. In Fig. 4(a), we started with the lowest-energy configuration of neutral g-C_4_N_3_ with a physisorbed CO_2_ molecule. Two electrons are then added to the neutral g-C_4_N_3_, and we examined the energy changes as the system relaxes to the 2 e^−^ negatively charged optimized state. In Fig. 4(b), we started with the lowest-energy configuration of the 2 e^−^ negatively charged g-C_4_N_3_ with a chemisorbed CO_2_ molecule. Two electrons are removed, and then the system is allowed to relax, forming a physisorbed CO_2_ molecule. When two extra electrons are introduced into g-C_4_N_3_, the interactions between the CO_2_ molecule and the 2 e^−^ negatively charged g-C_4_N_3_ are significantly larger than that with neutral g-C_4_N_3_, and the CO_2_ molecule spontaneously relaxes to chemisorption configuration. This process is exothermic by 1.08 eV without any energy barrier. On the other hand, when two extra electrons are removed from the 2 e^−^ negatively charged g-C_4_N_3_, the CO_2_ molecule spontaneously returns to the weakly bound state and desorbs from g-C_4_N_3_. This process is also exothermic by 1.39 eV without any energy barrier. Therefore, the CO_2_ storage/release processes on negatively charged g-C_4_N_3_ are reversible with fast kinetics, and can be easily controlled via adding/removing the extra electrons.

### The Effects of Charge Density on Single CO_2_ Capture on Negatively Charged g-C_4_N_3_ Nanosheets

To investigate the effects of charge density on CO_2_ capture on negatively charged g-C_4_N_3_, we investigated a CO_2_ adsorption on negatively charged g-C_4_N_3_ with different charge densities. Here, we defined the charge densities of g-C_4_N_3_ as follows
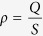
where *ρ*, *Q* and *S* are the charge densities of g-C_4_N_3_, the total charge and the surface area in 2 × 2 supercell, respectively. For g-C_4_N_3_, the surface area in 2 × 2 supercell can be calculated as 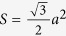 , where *a* is the lattice constant of 2 × 2 supercell.

**Figure 5:**
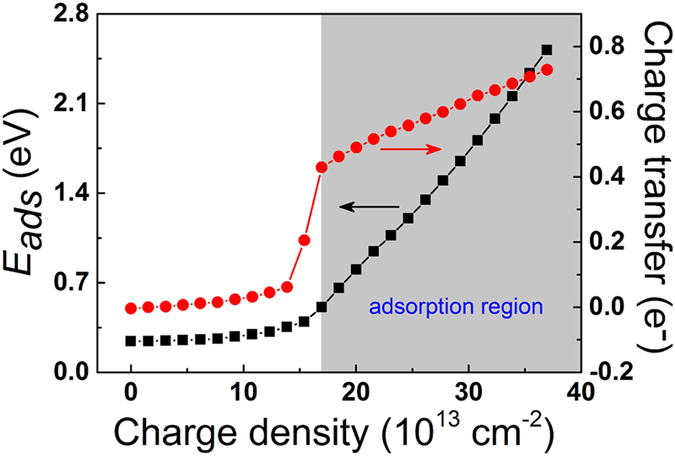
The adsorption energies of a CO_2_ on negatively charged g-C_4_N_3_ and the charge transfer between CO_2_ and g-C_4_N_3_ as functions of charge densities. The gray area indicates the adsorption region.

Figure 5 shows the adsorption energies of a CO_2_ on negatively charged g-C_4_N_3_ and the charge transfer between CO_2_ and g-C_4_N_3_ as functions of charge densities. For small charge density case (<13.9 × 10^13^ cm^−2^), the adsorption energy of CO_2_ is small (0.24 ~ 0.35 eV), and charge transfer between CO_2_ and g-C_4_N_3_ is less than 0.06 e^−^. When charge density is larger than 13.9 × 10^13^ cm^−2^, the adsorption energy of CO_2_ and the charge transfer from g-C_4_N_3_ to CO_2_ increase dramatically with increasing charge density, indicating the CO_2_ molecule can only adsorb on negatively charged g-C_4_N_3_ with large charge density. Considering the adsorption energies of CO_2_ on other high-performance adsorbents is 0.4−0.8 eV 6, we define the minimum charging density for CO_2_ capture on negatively charged g-C_4_N_3_ is about 17.0 × 10^13^ cm^−2^.

### CO_2_ Capture Capacity on Negatively Charged g-C_4_N_3_ Nanosheets

**Figure 6:**
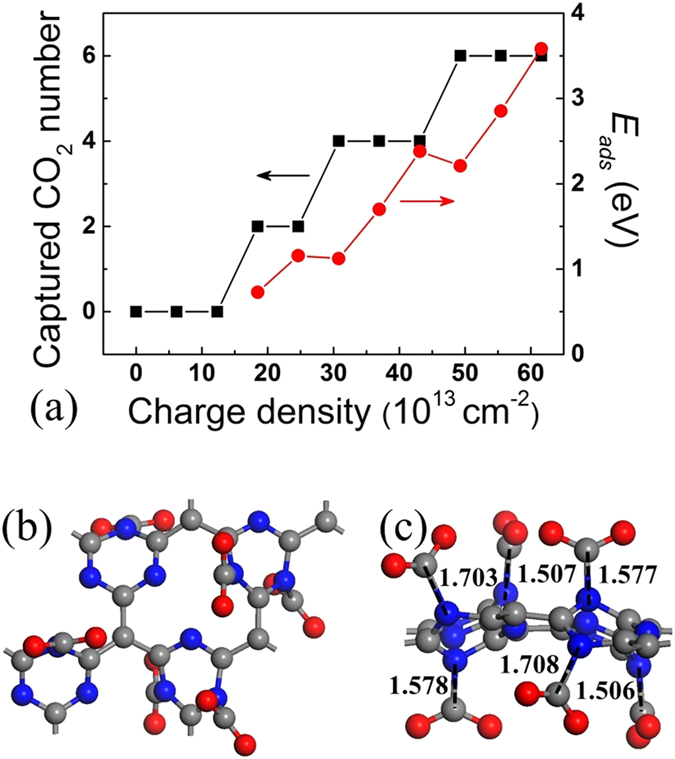
(**a**) The maximum number and the average adsorption energies of captured CO_2_ molecules on negatively charged g-C_4_N_3_ with different charge densities. (**b**) Top and (**c**) side views of the lowest-energy configuration of six CO_2_ molecules adsorbed on negatively charged g-C_4_N_3_ with charge density 61.7 × 10^13^ cm^−2^.

To estimate CO_2_ capture capacity on negatively charged g-C_4_N_3_, we studied the maximum number and the average adsorption energy of captured CO_2_ molecules on negatively charged g-C_4_N_3_ with different charge densities (Fig. 6(a)). Here, we determinate the maximum number of captured CO_2_ for each negatively charged g-C_4_N_3_ with different charge density by gradually increasing the number of CO_2_ molecules on negatively charged g-C_4_N_3_ until no more CO_2_ can be absorbed. The average adsorption energy of captured CO_2_ is calculated as the total adsorption energy divided by the maximum number of captured CO_2_. The results show that no CO_2_ molecules can be captured by negatively charged g-C_4_N_3_ with small charge density (≤12.3 × 10^13^ cm^−2^). As the charge density increase from 18.5 × 10^13^ to 61.6 × 10^13^ cm^−2^, the negatively charged g-C_4_N_3_ can capture two, four and six CO_2_ molecules with the average adsorption energy of captured CO_2_ molecules ranging from 0.72 to 3.58 eV. We note that a further increase in the number of CO_2_ molecules leads to some CO_2_ molecules moving far away from the adsorbent during the geometry optimization even if we further increase the charge density of g-C_4_N_3_. Therefore, we define six CO_2_ molecules in each 2 × 2 supercell (i.e. CO_2_ capture capacity 73.9 × 10^13^ cm^−2^ or 42.3 wt%) as the likely saturation CO_2_ capture coverage (Fig. 6(b,c)). It should be noted that surface defective sites such as N vacancies or un-condensed amino group could lower CO_2_ capture capacity. However, considering the high CO_2_ capture capacity of negatively charged g-C_4_N_3_, we believe this may nevertheless represent a feasible high-capacity CO_2_ capture material.

Interestingly, we note that the CO_2_ molecules do not all bind equally but the capture process occurs discretely two at a time. To further confirm this phenomenon, we put four CO_2_ molecules on neutral g-C_4_N_3_ and gradually increase the charge density of negatively charged g-C_4_N_3_ until four CO_2_ are all captured (corresponding lowest-energy configurations are shown in Figure S1 of the Supporting Information). Clearly, four CO_2_ are physically adsorbed on neural and 1 e^−^ negatively charged g-C_4_N_3_ (Figure S1(a,b), Supporting Information). On 2 e^−^ negatively charged g-C_4_N_3_, two CO_2_ are chemisorbed while other two CO_2_ are physisorbed on adsorbent (Figure S1(c), Supporting Information). When three electrons are introduced, all the CO_2_ are captured on 3 e^−^ negatively charged g-C_4_N_3_ (Figure S1(d), Supporting Information).

### CH_4_, H_2_ and N_2_ Adsorption on g-C_4_N_3_ Nanosheets

**Figure 7:**
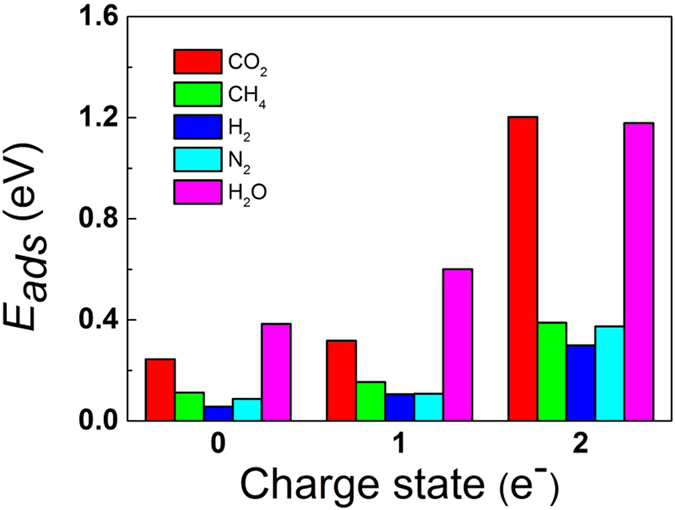
The adsorption energies of CO_2_, CH_4_, H_2_, N_2_ and H_2_O on neutral, 1 e^–^ and 2 e^–^ negatively charged g-C_4_N_3_.

CH_4_, H_2_, N_2_ are three types of gas mixtures that are currently most interesting for CO_2_ capture technologies, namely, postcombustion (predominantly CO_2_/N_2_ separation), natural gas sweetening (CO_2_/CH_4_), and precombustion (CO_2_/H_2_) capture^37^. In order to demonstrate the high selectivity of negatively charged g-C_4_N_3_ nanosheets for CO_2_ capture, we calculated the adsorption energies of CH_4_, H_2_ and N_2_ on neutral and negatively charged g-C_4_N_3_ and compared with those of CO_2_. In Fig. 7 we list the comparative adsorption energies of CO_2_, CH_4_, H_2_, and N_2_ on neutral, 1 e^−^ and 2 e^−^ negatively charged g-C_4_N_3_ (corresponding lowest-energy configurations are shown in Figure S2 of the Supporting Information). Clearly, the adsorptions of CH_4_, H_2_ and N_2_ on neutral, 1 e^−^ and 2 e^−^ g-C_4_N_3_ are all physical rather than chemical. The distance between the carbon atom of CH_4_ (the hydrogen atom of H_2_, the nitrogen atom of N_2_) and g-C_4_N_3_ is 3.157–3.159 (2.111–2.539, 2.865–3.236) Å, respectively. The adsorption energies of CH_4_, H_2_ and N_2_ on neutral, 1 e^−^ and 2 e^−^ g-C_4_N_3_ range from 0.06 to 0.39 eV. In contrast, although CO_2_ is physically adsorbed at neutral and 1 e^−^ g-C_4_N_3_ with small adsorption energy in the range from 0.24 to 0.32 eV, CO_2_ is tightly chemisorbed on 2 e^−^ g-C_4_N_3_ with large adsorption energy of 1.20 eV. The above comparisons demonstrate that negatively charged g-C_4_N_3_ has very high selectivity for capturing CO_2_ from CH_4_, H_2_ and/or N_2_ mixtures.

### Water Adsorption on g-C_4_N_3_ Nanosheets

Since water saturates most industrial gas streams, including flue gas, we also studied the adsorption energies of H_2_O on neutral and negatively charged g-C_4_N_3_ and compared with those of CO_2_, as shown in Fig. 7 (corresponding lowest-energy configurations are shown in Figure S2 of the Supporting Information). On neutral g-C_4_N_3_, both CO_2_ and H_2_O are physically adsorbed on adsorbents with small adsorption energies of 0.24 and 0.38 eV, respectively. On 1 e^−^ g-C_4_N_3_, the adsorption energy of CO_2_ slightly increases to 0.32 eV, while the adsorption energy of H_2_O significantly increases to 0.60 eV, which is twice as much as that of CO_2_. On 2 e^−^ g-C_4_N_3_, both CO_2_ and H_2_O are chemically adsorbed on adsorbents with large adsorption energies of 1.20 and 1.18 eV, respectively. These results indicate that the negatively charged g-C_4_N_3_ cannot selectively capture CO_2_ from a gas mixture with H_2_O present, and we should utilize some absorbent to eliminate water prior to CO_2_ adsorption. In fact, since the adsorption energy of H_2_O is twice as much as that of CO_2_ on 1 e^−^ g-C_4_N_3_, utilization of 1 e^−^ g-C_4_N_3_ to eliminate water prior to CO_2_ adsorption is one potentially viable approach. In this scanario, we could utilize g-C_4_N_3_ at lower voltage to eliminate water prior to a second stage of CO_2_ adsorption at slightly higher voltage.

## Discussion

In summary, we have shown that modification of the charge state of conductive g-C_4_N_3_ nanosheets provides an experimentally feasible approach for electrocatalytically switchable CO_2_ capture. Compared with other CO_2_ capture approaches, the process of CO_2_ capture/release occurs spontaneously without any energy barriers once extra electrons are introduced or removed, and these processes can be simply controlled and reversed by switching on/off the charging voltage. In addition, these negatively charged g-C_4_N_3_ nanosheets are highly selective for separating CO_2_ from mixtures with CH_4_, H_2_ and/or N_2_.

Good electrical conductivity and high electron mobility of the sorbent materials are prerequisite for electrocatalytically switchable CO_2_ capture. The aim of the present paper is to explore conductive and easily synthetic sorbent material as an experimentally feasible adsorbent for electrocatalytically switchable CO_2_ capture. These predictions may prove to be instrumental in searching for a new class of high-capacity CO_2_ capture materials with ideal thermodynamics and reversibility, and we hope that this work will stimulate further theoretical and experimental research in this direction.

## Methods

Our DFT calculations employed the linear combination of atomic orbital and spin-unrestricted method implemented in Dmol^3^ package^38^. The generalized gradient approximation (GGA) in Perdew-Burke-Ernzerhof (PBE) functional form^39^ together with an all-electron double numerical basis set with polarization function (DNP) were adopted. Since the standard PBE functional is incapable of giving an accurate description of weak interactions, we adopted a DFT+D (D stands for dispersion) approach with the Grimme’s vdW correction in our computations^40^. The real-space global cutoff radius was set to be 4.1 Å.

To study CO_2_ capture/release on g-C_4_N_3_ nanosheets, we employed 2 × 2 supercell with periodic boundary conditions in the *x*-*y* plane (Fig. 1(a)). The vacuum space was set to larger than 20 Å in the *z* direction to avoid interactions between periodic images. In geometry optimizations, all the atomic coordinates were fully relaxed up to the residual atomic forces smaller than 0.001 Ha/Å, and the total energy was converged to 10^−5^ Ha. The Brillouin zone integration was performed on a (6 × 6 × 1) Monkhorst-Pack k-point mesh^41^.

In order to investigate the gas adsorption on adsorbent, we defined the adsorption energy *E*_*ads*_ of CO_2_, CH_4_, H_2_ and N_2_ molecules on g-C_4_N_3_ as follows
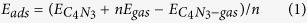
where 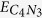, 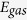, 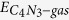, and 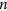 are the total energy of isolated g-C_4_N_3_ nanosheets, isolated gas molecule, g-C_4_N_3_ with the adsorbed gas, and number of gas molecules adsorbed on g-C_4_N_3_. According to this definition, a more positive adsorption energy indicates a stronger binding of the gas molecule to g-C_4_N_3_. The electron distribution and transfer mechanism are determined using the Mulliken method^42^.

## Additional Information

**How to cite this article**: Tan, X. *et al*. Conductive Graphitic Carbon Nitride as an Ideal Material for Electrocatalytically Switchable CO_2_ Capture. *Sci. Rep.* **5**, 17636; doi: 10.1038/srep17636 (2015).

## Supplementary Material

Supplementary Information
