# Supplementary material for: Conductive Graphitic Carbon Nitride as an Ideal Material for Electrocatalytically Switchable CO2 Capture
Source: Sci Rep. 2015 Dec 1;5:17636. doi: 10.1038/srep17636 (PMC4664948; doi:10.1038/srep17636)
Supplement: Supplementary Information [file srep17636-s1.doc]

Supplementary information

**Conductive Graphitic Carbon Nitride as an Ideal Material for Electrocatalytically Switchable CO_2_ Capture**

Xin Tan, Liangzhi Kou, Hassan A. Tahini, Sean C. Smith[[1]](#footnote-2)*

Integrated Materials Design Centre (IMDC), School of Chemical Engineering, UNSW Australia, Sydney, NSW 2052, Australia


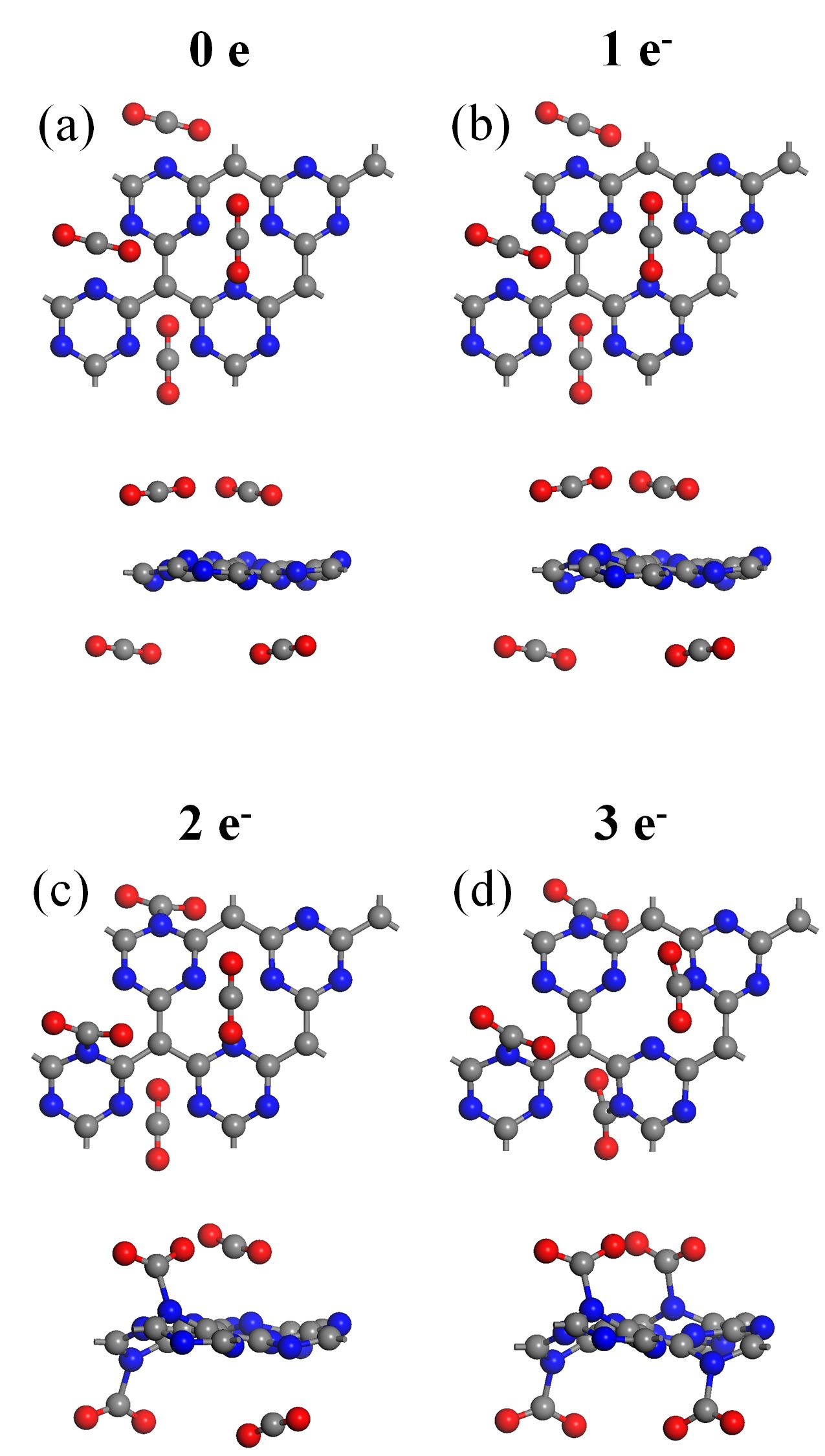


**Supplementary Figure S1.** Top (upper) and side (lower) views of the lowest-energy configurations of four CO_2_ molecules on (a) neutral, (b) 1 e^-^, (c) 2 e^-^ and (d) 3 e^-^ negatively charged g-C_4_N_3_.


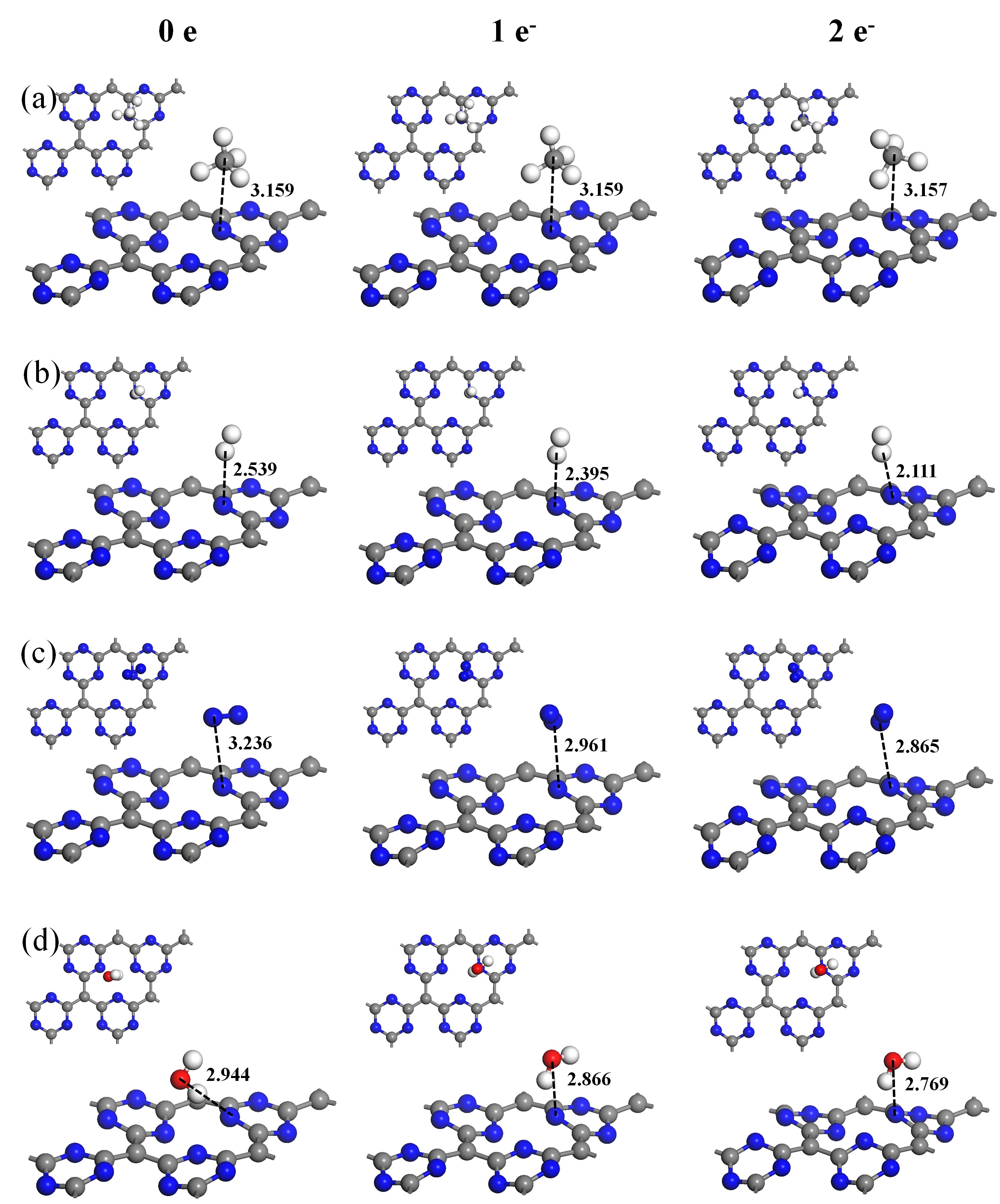


**Supplementary Figure S2.** Top and side views of the lowest-energy configurations of (a) CH_4_, (b) H_2_, (c) N_2_ and (d) H_2_O absorbed on neutral, 1 e^-^ and 2 e^-^ negatively charged g-C_4_N_3_.

1. ^^ Corresponding author: sean.smith@unsw.edu.au [↑](#footnote-ref-2)
